# Prognostic value of the Stanniocalcin-2/PAPP-A/IGFBP-4 axis in ST-segment elevation myocardial infarction

**DOI:** 10.1186/s12933-018-0710-3

**Published:** 2018-04-30

**Authors:** Germán Cediel, Ferran Rueda, Claus Oxvig, Teresa Oliveras, Carlos Labata, Oriol de Diego, Marc Ferrer, M. Cruz Aranda-Nevado, Judith Serra-Gregori, Julio Núñez, Cosme García, Antoni Bayes-Genis

**Affiliations:** 1 Heart Institute, Hospital Universitari Germans Trias i Pujol, Carretera de Canyet s/n, Badalona, 08916 Barcelona, Spain; 2 Department of Medicine, CIBERCV, Autonomous University of Barcelona, Barcelona, Spain; 3 Department of Molecular Biology and Genetics, Aarhus University, Aarhus, Denmark; 4 Cardiology Department, Hospital Clínico Universitario, INCLIVA, Departamento de Medicina, CIBERCV Universitat de València, Valencia, Spain

**Keywords:** STEMI, Prognosis, Stanniocalcin-2, PAPP-A, IGFBP-4

## Abstract

**Objective:**

The aim of the present study was to evaluate the prognostic value of the Stanniocalcin-2/PAPP-A/IGFBP-4 axis in patients with ST-segment elevation myocardial infarction (STEMI).

**Methods:**

Observational cohort study performed in 1085 consecutive STEMI patients treated with early reperfusion between February 2011 and August 2014. Stanniocalcin-2, PAPP-A, and IGFBP-4 were measured using state-of-the art immunoassays. The primary outcome was the composite endpoint of all-cause mortality and readmission due to heart failure (HF).

**Results:**

Median follow-up was 3.3 years (IQR 1.0–3.7), during which 176 patients (16.2%) presented a composite endpoint. Multivariable cox regression analysis revealed that Stanniocalcin-2 (HR 2.06; 95% CI 1.13–3.75; p = 0.018), IGFBP-4 (HR 1.73; 95% CI 1.14–2.64; p = 0.010), Killip–Kimball class III–IV (HR 1.40; 95% CI 1.13–1.74; p = 0.002), NT-ProBNP (HR 1.21; 95% CI 1.07–1.37; p = 0.002), age (HR 1.06; 95% CI 1.04–1.08; p < 0.001) and left ventricular ejection fraction (HR 0.97; 95% CI 0.95–0.98; p < 0.001) were independent predictors of the composite endpoint. A model containing Stanniocalcin-2 and IGFBP-4 on top of clinical variables significantly improved C-index discrimination (p = 0.036). Stanniocalcin-2 was also identified as independent predictor of all-cause mortality (HR 2.23; 95% CI 1.16–4.29; p = 0.017) and readmission due to HF (HR 3.42; 95% CI 1.22–9.60; p = 0.020).

**Conclusions:**

In STEMI patients, Stanniocalcin-2 and IGFBP-4 emerged as independent predictors of all-cause death and readmission due to HF. The Stanniocalcin-2/PAPP-A/IGFBP-4 axis exhibits a significant role in STEMI risk stratification.

## Background

Patients with acute ST-segment elevation myocardial infarction (STEMI) are at considerable risk for cardiovascular complications and death despite remarkable advances in non-invasive and invasive treatment [1]. Early risk stratification is therefore important for the assessment of prognosis as well as to guide adequate secondary prevention treatment. In this setting, the value of biomarkers reflecting different disease pathways is under scrutiny.

**Fig. 1:**
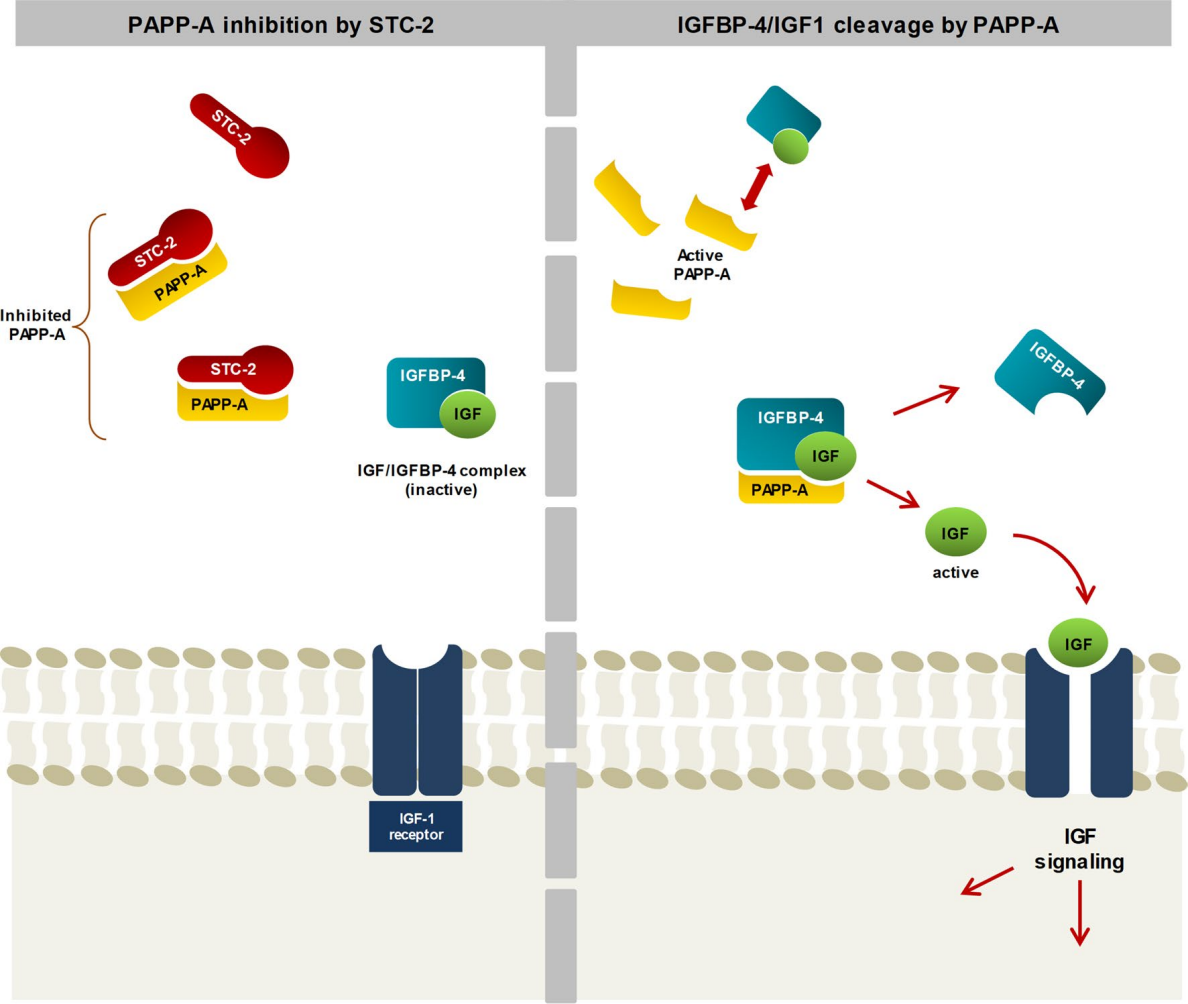
Schematic of the Stanniocalcin-2/PAPP-A/IGFBP-4 axis. Left: Stanniocalcin-2 inhibits PAPP-A cleavage of IGFBP-4 thereby resulting in decreased levels of free IGF, and consequently decreased IGF signaling. Right: in the absence of Stanniocalcin-2, PAPP-A cleaves IGFBP-4, resulting in liberation of free IGF. Because free IGF can bind its receptor, IGF signaling is then induced. *STC-2* Stanniocalcin-2, *PAPP-A* pregnancy-associated plasma protein-A, *IGFBP-4* insulin-like growth factor binding protein 4, *IGF* insulin-like growth factor

Pregnancy-associated plasma protein-A (PAPP-A), a high molecular weight and zinc-binding metalloproteinase, has been regarded a candidate marker in cardiovascular disease and vulnerable plaque [2, 3]. PAPP-A is an important regulatory protein in cell proliferation and the development of atherosclerosis [4, 5, 9]. PAPP-A is specific for three insulin-like growth factor binding proteins (IGFBP-2, -4, and -5) and functions intimately with IGFBP-4, which is a key regulator of insulin-like growth factor (IGF) bioactivity [6–8]. Recently, Stanniocalcin-2 was identified as a novel modulator of IGF bioavailability due to its potential to inhibit the proteolytic activity of PAPP-A [9] (Fig. 1). In atherosclerotic plaques, PAPP-A and Stanniocalcin-2 are abundantly expressed [10]. Collectively, the Stanniocalcin-2/PAPP-A/IGFBP-4 axis regulates local IGF bioavailability and signaling with potential biological implications in cardiovascular disease [8]. Despite recent studies showing that PAPP-A and IGFBP-4 are potentially important biomarkers for the prediction of adverse outcomes in patients with acute coronary syndrome [11, 12], the evidence is scarce in contemporary-treated STEMI patients promptly reperfused, and to date, there are no reports on the prognostic value of Stanniocalcin-2 in this population.

Accordingly, the present study was designed to evaluate the prognostic value of the Stanniocalcin-2/PAPP-A/IGFBP-4 axis in a consecutive STEMI population exclusively treated by primary percutaneous coronary intervention (PPCI).

## Methods

### Study design and population

Prospective observational study that included consecutive STEMI patients treated with PPCI between February 22, 2011, and November 11, 2014. Our institute is a tertiary university hospital that serves a population of ~ 850,000 inhabitants, mainly distributed among 4 urban areas located 2, 7, 20, or 45 km from our PPCI-capable unit. The diagnosis of STEMI was established when patients presented with chest pain and an electrocardiogram showed ST-segment elevation in two or more contiguous leads (elevation was defined as a minimum of 0.1 mV in the frontal leads and 0.2 mV in the precordial leads) or with a left bundle branch block (new onset or indeterminate chronology) that evolved within 6 h [13, 14]. Baseline demographics and clinical data were recorded during hospital admission. Blood samples were obtained upon coronary angiography and were processed in a central laboratory for biomarker measurements. The left ventricular ejection fraction (LVEF) was assessed within the first 24 h of admission with echocardiography (Agilent Sonos 5500-Philips and ie33-Philips) using the Simpson method.

### Study outcome and follow-up

The main clinical outcome was the composite of all-cause mortality and readmission due to heart failure (HF). Follow-up events were obtained from searching the patients’ electronic clinical records, death registers or by contacting the patient’s relatives. For patients with recurrent events, the time to the first event was recorded. All participants gave their informed consent, and this study was performed in compliance with the Helsinki Declaration, and was approved by the local Ethics Committee.

### Biomarker assays

High sensitive troponin T (hs-TnT) was measured with an electrochemiluminescence immunoassay (ultrasensitive troponin T method, ref 05092744 190; Roche Diagnostics) performed on a cobas e601 analyzer (Roche Diagnostics). The analytic performance of this assay has been validated [15]. As described by the manufacturer (ref 05092744 190; Roche Diagnostics), the 99th percentile for normal was 14 ng/L and the functional sensitivity (limit of quantification with a coefficient of variation of < 10%) was 13 ng/L. NT-proBNP levels were determined using an immuno-electrochemiluminescence assay and the Modular Analytics E170 (Roche Diagnostics Inc., Indianapolis, IN). This assay has < 0.001% cross-reactivity with bioactive BNP, and the assay had inter-run coefficients of variation ranging from 0.9 to 5.5%. Immunoassays for Stanniocalcin-2, PAPP-A, and intact IGFBP-4 were from AnshLabs, Webster, TX, USA. Stanniocalcin-2 levels were measured using an enzyme linked immunosorbent assay (AL-143) with a limit of detection of 0.033 ng/mL; PAPP-A was measured with an enzyme linked immunosorbent assay (picoPAPP-A, AL-101), and as described by the manufacturer the limit of detection is 0.037 ng/mL; intact IGFBP-4 was measured with an enzyme linked immunosorbent assay (AL-124) with a limit of detection of 0.669 ng/mL.

### Statistical analysis

Categorical variables are presented as number and percentage. Continuous variables are reported as median and interquartile range. Non-normally distributed variables were log-transformed prior to statistical analyses. A composite endpoint including all events was used for comparison of baseline characteristics. Patients with or without events during follow-up were compared using the Chi squared test or Fisher’s exact test for categorical variables and the Wilcoxon rank sum test for continuous variables. Survival analyses were performed using Cox proportional hazards models (using the backward elimination method). Hazard ratios (HRs) with 95% confidence intervals (CIs) are reported. The following variables were incorporated into the regression models: age, sex, history of hypertension, diabetes mellitus, cerebrovascular disease, HF, myocardial infarction, previous coronary artery disease, Killip–Kimball class category, LVEF, estimated glomerular filtration rate, low-density lipoprotein, PAPP-A, Stanniocalcin-2, IGFBP-4, hs-TnT and NT-ProBNP. For each end-point, the interactions between the Stanniocalcin-2, PAPP-A and IGFBP-4 was explored without finding statistically significance in any of them. To evaluate the proportional hazards assumption, Schoenfeld residuals were assessed. We used a multivariable competing risk model to obtain the HRs for readmission due to HF, considering death as a competing event. We plotted log-derived hazards for major cardiovascular events relative to baseline Stanniocalcin-2 and IGFBP-4 values using fractional polynomials. To assess the discrimination benefit of adding PAPP-A, IGFBP-4 and Stanniocalcin-2 to a clinical model, Harrell’s C statistics was used; calibration was assessed using the Royston modification of Nagelkerke’s *R*^2^ statistic for proportional hazards models, and continuous net reclassification improvement (NRI) was used for reclassification prediction. Statistical analysis was performed using STATA V.13.0 (College Station, Texas, USA). A p value of < 0.05 was considered significant.

## Results

### Baseline characteristics

From a total of 1163 consecutive patients admitted during the study period, 1085 (93.3%) had all biomarkers measured and were finally included in the study. Median follow-up was 3.3 years (IQR 1.0–3.7), during which 176 patients (16.2%) presented a composite endpoint (136 deaths and 60 readmissions due to HF). Table 1 shows the baseline characteristics of patients included in the study. Patients with events were more likely to be women and older and were more likely to have a history of arterial hypertension, diabetes mellitus, cerebrovascular disease, peripheral arterial disease, HF and myocardial infarction. Patients who presented the composite endpoint exhibited greater disease severity (Killip–Kimball class III or IV, 25.0% vs. 3.5%; p < 0.001) worse LVEF (45% vs. 54%; p < 0.001) and more prevalent extensive coronary artery disease (3-vessel disease) than patients without events during follow-up (32.4% vs. 17.1%; p < 0.001).

**Table 1:** Baseline characteristics of the studied cohort relative to the presence of events

Variable	All patients (n = 1085)	Without events (n = 909)	With events (n = 176)	p value
Age (years)	62 (52–73)	59 (51–69)	76 (66–81)	< 0.001
Female	241 (22.2)	187 (20.6)	54 (30.7)	0.003
Hypertension	598 (55.1)	473 (52.0)	125 (71.0)	0.001
Diabetes mellitus	276 (25.4)	208 (22.9)	68 (38.6)	< 0.001
Dyslipidemia	645 (59.5)	550 (60.5)	95 (54.0)	0.106
Cerebrovascular disease	69 (6.4)	42 (4.6)	27 (15.3)	< 0.001
Peripheral arterial disease	77 (7.1)	57 (6.3)	20 (11.4)	0.016
Heart failure	18 (1.7)	7 (0.8)	11 (6.3)	< 0.001
Coronary artery disease	236 (21.8)	188 (20.7)	48 (27.3)	0.052
Myocardial infarction	103 (9.5)	76 (8.4)	27 (15.3)	0.004
Killip–Kimball I–II	1009 (93.0)	877 (96.5)	132 (75.0)	< 0.001
Killip–Kimball III–IV	76 (7.0)	32 (3.5)	44 (25.0)	< 0.001
BMI (kg/m^2^)	27.3 (24.8–29.9)	27.2 (24.8–29.8)	27.6 (24.3–30.0)	0.944
Hs-TnT, peak (ng/L)	2752 (1021–6057)	2643 (934–5590)	4375 (1357–91,485)	< 0.001
NT-ProBNP, peak (ng/L)	1003 (395–2421)	827 (356–1707)	3461 (1569–7396)	< 0.001
LVEF (%)	52 (45–58)	54 (47–59)	45 (38–53)	< 0.001
Main epicardial coronary arteries > 70% stenosis				
1	564 (52.0)	493 (54.2)	71 (40.3)	0.001
2	287 (26.5)	253 (27.8)	34 (19.3)	0.019
3	212 (19.5)	155 (17.1)	57 (32.4)	< 0.001
Left main ≥ 50% stenosis	40 (3.7)	25 (2.8)	15 (8.5)	< 0.001

Data represent the number (%) or median (interquartile range). Wilcoxon Rank-sum test was used for comparisons of continuous variables

*PCI* percutaneous coronary intervention, *BMI* body mass index, *hs-TnT* high-sensitivity troponin T, *LVEF* left ventricular ejection fraction

An unadjusted analysis showed that patients with events had a significantly higher median (IQR) hs-TnT levels (4375 [1357–91,485] vs. 2643 [934–5590] ng/L; p < 0.001) and higher median NT-ProBNP levels (3461 [1569–7396] vs. 827 [356–1707] ng/L; p < 0.001). Likewise, median levels of PAPP-A (12.3 [5.4–28.6] vs. 10.1 [4.2–22.5] µg/L; p = 0.019), Stanniocalcin-2 (19.2 [15.3–24.3] vs. 17.5 [14.7–21.2] µg/L; p < 0.001) and intact (uncleaved) IGFBP-4 (36.8 [27.6–48.1] vs. 29.5 [23.0–36.7] µg/L; p < 0.001) were also significantly higher in patients who presented the composite endpoint compared with those without events.

### Predictors of long-term outcomes

**Fig. 2:**
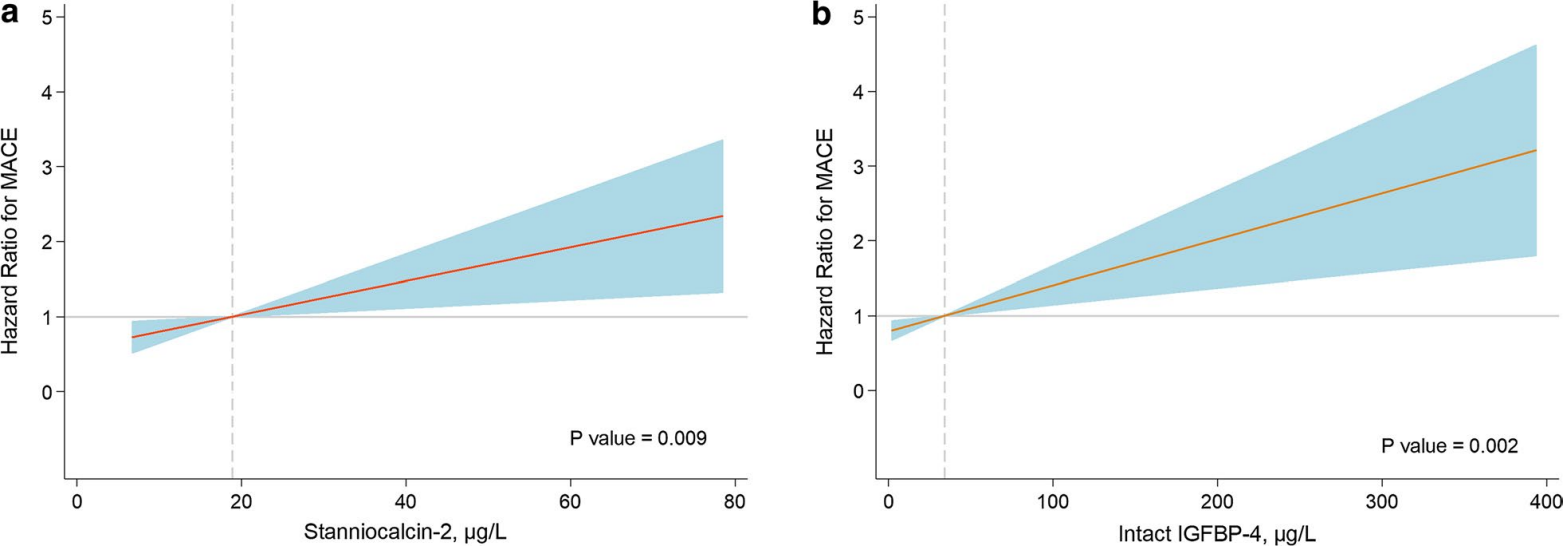
Multivariable analyses for MACE. **a** HR gradient (shaded area = 95% CI), with median Stanniocalcin-2 value (17.8 µg/L) as reference. **b** HR gradient (shaded area = 95% CI), with median IGFBP-4 value (30.5 µg/L) as reference. Both analyses adjusted for age, sex, history of arterial hypertension, diabetes mellitus, heart failure, myocardial infarction, glomerular filtration rate, killip–kimball class and left ventricular ejection fraction. *CI* confidence interval, *HR* hazard ratio, *MACE* major adverse cardiovascular events

Multivariable Cox proportional-hazard regression models showed that Stanniocalcin-2 (HR 2.06; 95% CI 1.13–3.75; p = 0.018), IGFBP-4 (HR 1.73; 95% CI 1.14–2.64; p = 0.010), Killip–Kimball class III or IV (HR 1.40; 95% CI 1.13–1.74; p = 0.002), NT-ProBNP (HR 1.21; 95% CI 1.07–1.37; p = 0.002), age (HR 1.06; 95% CI 1.04–1.08; p < 0.001) and LVEF (HR 0.97; 95% CI 0.95–0.98; p < 0.001) were independent predictors of the composite endpoint during follow-up. Further, Stanniocalcin-2 was identified as independent predictor of all-cause mortality (HR 2.23; 95% CI 1.16–4.29; p = 0.017) and readmission due to HF (HR 3.42; 95% CI 1.22–9.60; p = 0.020) (Table 2). There was no significant association between PAPP-A or hs-TnT levels and the composite endpoint (Table 3). The relationship between Stanniocalcin-2 and IGFBP-4 levels as continuous variables and the relative risk for the composite endpoints is shown in Fig. 2.

**Table 2:** Multivariable Cox regression analyses for the composite endpoint of all-cause mortality and readmission due to heart failure

	HR (95% CI)	p value
Composite endpoint		
Stanniocalcin-2^a^	2.06 (1.13–3.75)	0.018
IGFBP-4^a^	1.73 (1.14–2.64)	0.010
Killip–Kimball class III–IV	1.40 (1.13–1.74)	0.002
NT-ProBNP^a^	1.21 (1.07–1.37)	0.002
Age	1.06 (1.04–1.08)	< 0.001
LVEF	0.97 (0.95–0.98)	< 0.001
All-cause mortality		
Stanniocalcin-2^a^	2.23 (1.16–4.29)	0.017
Killip–Kimball class II–IV	1.64 (1.30–2.08)	< 0.001
NT-ProBNP^a^	1.18 (1.02–1.36)	0.028
Age	1.07 (1.05–1.10)	< 0.001
LVEF	0.97 (0.95–0.99)	0.002
Readmission due to heart failure		
Stanniocalcin-2^a^	3.42 (1.22–9.60)	0.020
Prior myocardial infarction	2.85 (1.32–6.16)	0.008
Diabetes mellitus	2.36 (1.22–4.58)	0.011
Female sex	2.21 (1.15–4.28)	0.018
Age	1.04 (1.01–1.07)	0.005
LVEF	0.94 (0.92–0.97)	< 0.001

*IGFBP-4* insulin-like growth factor binding protein-4, *LVEF* left ventricular ejection fraction

^a^HRs (95% CIs) are reported per 1-SD increment of natural log-transformed plasma levels

**Table 3:** Univariable and multivariable Cox regression analyses for the composite endpoint of all-cause mortality and readmission due to heart failure

	Composite endpoint		All-cause mortality		Readmission due to HF	
	HR (95% CI)	p	HR (95% CI)	p	HR (95% CI)	p
PAPP-A						
Univariable	1.25 (1.09–1.42)	0.001	1.30 (1.12–1.51)	0.001	1.08 (0.85–1.38)	0.525
Adjusted	1.13 (0.94–1.35)	0.185	1.10 (0.90–1.35)	0.340	0.94 (0.65–1.35)	0.729
Stanniocalcin-2						
Univariable	2.90 (1.81–4.64)	< 0.001	2.52 (1.47–4.29)	0.001	5.37 (2.35–12.26)	< 0.001
Adjusted	2.06 (1.13–3.75)	0.018	2.23 (1.16–4.29)	0.017	3.42 (1.22–9.60)	0.020
IGFBP-4						
Univariable	3.29 (2.47–4.39)	< 0.001	3.18 (2.30–4.39)	< 0.001	2.69 (1.83–3.97)	< 0.001
Adjusted	1.73 (1.14–2.64)	0.010	1.44 (0.85–2.44)	0.170	0.82 (0.42–1.60)	0.562
Hs-TnT						
Univariable	1.48 (1.32–1.65)	< 0.001	1.44 (1.27–1.63)	< 0.001	1.64 (1.36–1.98)	< 0.001
Adjusted	1.07 (0.91–1.25)	0.424	1.04 (0.87–1.25)	0.665	1.00 (0.81–1.26)	0.959
NT-ProBNP						
Univariable	1.69 (1.54–1.84)	< 0.001	1.66 (1.50–1.84)	< 0.001	1.68 (1.43–1.97)	< 0.001
Adjusted	1.21 (1.07–1.37)	0.002	1.18 (1.02–1.36)	0.028	1.24 (0.92–1.68)	0.157

All HRs (95% CIs) are reported per 1-SD increment of natural log-transformed plasma levels

*hs-TnT* high-sensitivity troponin T, *PAPP-A* pregnancy-associated plasma protein-A, *IGFBP-4* insulin-like growth factor binding protein-4

Table 4 shows calibration, discrimination and reclassification metrics to evaluate the incremental value of the Stanniocalcin-2/PAPP-A/IGFBP-4 axis components over a clinical model for prediction of events during follow-up. For the composite endpoint, the addition of each biomarker alone to the clinical model did not significantly improve discrimination; however, a model containing Stanniocalcin-2 and IGFBP-4 on top of clinical variables improved discrimination, with an increase in the C-index from 0.815 to 0.826 (p = 0.036) with a higher Nagelkerke’s R2. The associated continuous NRI was 0.140 95% CI [0.004–0.428], corresponding to 6.2% patients reclassified. For the prediction of all-cause mortality or HF readmission, the addition of these biomarkers to the clinical model did not significantly improve performance metrics (Table 4).

**Table 4:** Performance of Models for the composite endpoint of all-cause mortality and readmission due to heart failure

Marker	C-statistic (p value)	Nagelkerke’s R^2^	cNRI	Reclassification
Composite endpoint				
Clinical model (CM)	0.815	0.645		
CM + Stanniocalcin-2	0.821 (p = 0.100)	0.660	0.125 (− 0.053–0.363)	3.8% (0.9–6.9%)
CM + IGFBP-4	0.823 (p = 0.113)	0.671	0.270 (0.064–0.431)	5.0% (2.2–8.9%)
CM + Stanniocalcin-2 + IGFBP-4	0.826 (p = 0.036)	0.677	0.176 (0.001–0.420)	6.2% (3.1–9.6%)
All-cause mortality				
Clinical model (CM)	0.807	0.636		
CM + Stanniocalcin-2	0.808 (p = 0.555)	0.646	0.129 (− 0.129–0.330)	2.4% (0.3–4.8%)
CM + IGFBP-4	0.814 (p = 0.241)	0.662	0.248 (0.001–0.412)	3.7% (1.2–6.6%)
CM + Stanniocalcin-2 + IGFBP-4	0.814 (p = 0.204)	0.664	0.185 (− 0.041–0.433)	4.1% (1.6–7.0%)
Readmission due to heart failure				
Clinical model (CM)	0.861	0.802		
CM + Stanniocalcin-2	0.872 (p = 0.211)	0.825	0.256 (− 0.129–0.653)	1.31% (0.2–3.0%)
CM + IGFBP-4	0.861 (p = 0.103)	0.806	0.114 (− 0.285–0.446)	0.4% (0.0–1.9%)
CM + Stanniocalcin-2 + IGFBP-4	0.869 (p = 0.413)	0.823	0.239 (− 0.148–0.611)	1.6% (0.3–3.1%)

Clinical model includes: age, sex, history of arterial hypertension, diabetes mellitus, heart failure, myocardial infarction, glomerular filtration rate and left ventricular ejection fraction

*IGFBP-4* insulin-like growth factor binding protein-4

## Discussion

### Main findings

This observational prospective study investigated the association between the Stanniocalcin-2/PAPP-A/IGFBP-4 axis and adverse events in a cohort of unselected patients with STEMI. After adjusting for clinical predictors, Stanniocalcin-2 and IGFBP-4 were significant and independent predictors of the composite endpoint and Stanniocalcin-2 emerged also as an independent predictor of all-cause mortality and readmission due to HF. These findings suggest that the Stanniocalcin-2/PAPP-A/IGFBP-4 axis is of value in the risk stratification of STEMI patients.

### Stanniocalcin-2/PAPP-A/IGFBP-4 axis and long-term outcomes

PAPP-A is a large, zinc binding proteinase produced by different cell types, including fibroblasts, vascular smooth muscle cells, and male and female reproductive tissues, and is widely used in prenatal screening [16]. PAPP-A shows proteolytic activity towards IGFBP-2, IGFBP-4 and IGFBP-5. It functions intimately with IGFBP-4 and its cleavage that occurs in close proximity to the IGF1 receptor allows dissociation of bound active IGF, increasing IGF signaling through receptor stimulation [17]. Recently, Stanniocalcin-2 has been reported to be a potent inhibitor of PAPP-A proteolytic activity. Stanniocalcin-2 binds covalently to PAPP-A to completely eliminate its activity toward IGFBP-4 and hence PAPP-A-mediated IGF signaling [9]. Thus, the Stanniocalcin-2/PAPP-A/IGFBP-4 axis regulates local IGF bioavailability and signaling (Fig. 1) stimulating cell proliferation and promoting macrophage activation, low-density lipoprotein uptake and release of pro-inflammatory cytokines [18]. This axis represents an intriguing disease pathway of increasing interest.

The first component of the axis that was related to atherosclerosis was PAPP-A. In 2001, Bayes-Genis et al. [2] first described that circulating PAPP-A levels are upregulated in acute coronary syndrome (ACS), suggesting that PAPP-A might be a useful biomarker of plaque instability, Since then, several studies have shown that elevations of PAPP-A are associated with recurrent cardiovascular events in patients with non-ST segment elevation-ACS [19, 20] and in patients with stable cardiovascular disease and indications for cardiac catheterization [21] Subsequent studies showed that heparin treatment, common among ACS patients, induces a drastic increase in serum PAPP-A within few minutes, from the arterial wall and not atherosclerotic plaques, shedding doubt on PAPP-A as a reliable biomarker of adverse events in ACS [22–24]. In this study, PAPP-A was measured immediately before catheterization but after heparin administration.

Consequently, different assays that measure IGFBP-4 as the cleaved substrate of active PAPP-A have been developed, on the basis that it may be reflective of PAPP-A enzymatic activity and may reflect plaque burden serving as marker of atherosclerosis [25]. Studies of IGFBP-4 as a cardiac risk marker have shown controversial results that may be justified by the differences in the study population, and the clinical setting where the IGFBP-4 was studied. In patients with stable cardiovascular disease, Schulz et al. [26] found that CT- and NT-IGFBP4 levels failed to predict any long-term outcomes, whereas Postnikov et al. [25] found that in patients with symptoms of myocardial ischemia but without ST-segment elevation, both NT- and CT-IGFBP-4 were strong predictors of major adverse cardiac events and more recently, Hjortebjerg et al. [12] found that IGFBP-4 fragments were associated with increased risk of all-cause mortality, cardiovascular mortality and major adverse cardiac events in patients with STEMI. Nonetheless, our results support the evidence that in STEMI patients, IGFBP-4 is associated with an increased risk of adverse events during long term follow-up and favor the hypothesis that its prognostic ability is notable in patients in the acute phase of a myocardial infarction, even in the contemporary setting of routine primary PCI. The prognostic value of intact IGFBP-4 shown in our study, differs from what was previously observed by Postnikov [25] and Hjortebjerg [12], where measurements of full-length IGFBP4 were not predictive for MACE and intact IGFBP-4 did not perform better than C reactive protein or peak troponin I for any end point, respectively. These differences may in part be explained by the different immunoassays used in the studies. Further, previous in vitro and in vivo studies have shown that intact IGFBP-4 exerts mainly inhibitory effects on IGF functionality [27]. Thus, we hypothesized that in the setting of ACS, the increased PAPP-A proteolytic activity with secondary augmented IGF bioavailability leads to increased serum levels of intact IGFBP-4 as a regulatory response; this is likely the reason higher levels of intact IGFBP-4 are associated with the composite endpoint in this clinical setting.

Due to the recent discovery of Stanniocalcin-2 as a novel proteinase inhibitor of PAPP-A, we considered that it could also reflect PAPP-A enzymatic activity, with potential prognostic implications in the setting of acute coronary syndrome similarly to IGFBP-4. In this study we found that elevated levels of Stanniocalcin-2 are significantly associated with increased risk of all-cause mortality and readmission due to HF in STEMI patients. We hypothesized that Stanniocalcin-2 levels are regulated by IGF bioavailability: in a high PAPP-A setting with subsequent cleavage of the IGFBP-4/IGF1 complex and increased IGF signaling (e.g. in ACS where PAPP-A is abundantly expressed in both eroded and ruptured plaques), higher levels of Stanniocalcin-2 arise as a compensatory response. It should be emphasized that Stanniocalcin-2 upregulation unrelated to IGF signaling is also possible; this upregulation might be also activated in response to oxidative stress and hypoxia after coronary artery occlusion in a similar way to what had been demonstrated in the animal model after cerebral artery occlusion [28] or in the setting of hypoxic tumor microenvironment [29].

At the turn of the century, some studies suggested that low IGF-I levels were associated with increased ischemic heart disease [30] and that IGF-1 protects against endothelial dysfunction, atherosclerotic plaque development, metabolic syndrome, clinical instability, and ischemic myocardial damage [31]. By contrast, more recent research in preclinical models showed that smooth muscle cell proliferation and migration in neointimal hyperplasia was markedly reduced in the absence of PAPP-A [32] and that PAPP-A substrate binding site inhibition reduces atherosclerotic plaque burden [33]; supporting this theory, and specifically in the population of STEMI patients, our results suggest that within the axis, elevated levels of Stanniocalcin-2 and intact IGFBP-4 can be interpreted as a regulatory response to high PAPP-A proteolytic activity, The specific mechanisms in STEMI patients warrant further investigation.

In the full spectrum of ACS, the role of biomarkers for actionable risk stratification has proved useful in patients with unstable angina or non-STEMI. As PPCI is the cornerstone of STEMI treatment, the main interest of such biomarkers in this population, lies in their ability to provide long-term prognostication (focusing on the population of hospital survivors). Remarkably, we found superior predictive ability for Stanniocalcin-2 and IGFBP-4 in comparison to previous validated biomarkers such as high-sensitivity cardiac troponin, which may no longer provide added value in STEMI risk-assessment [34]. It is possible that in the era of routine PPCI with subsequent decrease in infarct size, novel biomarkers representing different and specific pathways may emerge as useful risk stratification tools. Our findings support the hypothesis that the Stanniocalcin-2/PAPP-A/IGFBP-4 axis is of remarkable importance in the vascular response to injury and in atherosclerosis and plays an important role in the risk stratification of STEMI patients. Accordingly, Stanniocalcin-2 and IGFBP-4 may become useful prognostic biomarkers for increased risk of adverse outcomes in STEMI patients; indeed, their prognostic value is additive to other traditional clinical risk factors in refining clinical decision making.

### Limitations

Some limitations of our study should be acknowledged to aid in data interpretation. This is a single centre, prospective study design, and the results must be interpreted in that light. Samples were collected at baseline with no measurement beyond; therefore, we are not able to evaluate dynamic changes in variables over time. The cause of death for patients in the study was not investigated. Despite these limitations, our findings were representative of a broad range of unselected patients with STEMI, which reflect a real-life clinical scenario in our daily practice.

## Conclusions

In contemporary-treated STEMI patients, Stanniocalcin-2 and IGFBP-4 were strong predictors of mortality and HF readmission and may become valuable cardiovascular biomarkers, combined with traditional clinical predictors, for identifying high-risk STEMI patients. The Stanniocalcin-2/PAPP-A/IGFBP-4 axis is a new disease pathway with a significant role in the risk stratification of STEMI patients. Further research is needed to validate our data.

## Abbreviations

ACS: acute coronary syndrome
CI: confidence intervals
HF: heart failure
HR: hazard ratio
hs-TnT: high sensitive troponin T
IGF: insulin-like growth factor
IGFBP: insulin-like growth factor binding protein
LVEF: left ventricular ejection fraction
NRI: net reclassification improvement
PAPP-A: pregnancy-associated plasma protein-A
PPCI: primary percutaneous coronary intervention
STEMI: ST-segment elevation myocardial infarction
